# Beta-trace protein concentrations at the blood-cerebrospinal fluid barrier – acute phase affects protein status

**DOI:** 10.17179/excli2021-4148

**Published:** 2021-09-27

**Authors:** Ingrid Lafer, Simon Michaelis, Christopher Schneider, Andreas Baranyi, Wolfgang J. Schnedl, Sandra Holasek, Sieglinde Zelzer, Tobias Niedrist, Andreas Meinitzer, Dietmar Enko

**Affiliations:** 1 Department of Internal Medicine, General Hospital Hochsteiermark, Mürzzuschlag, Austria; 2 Institute of Clinical Chemistry and Laboratory Medicine, General Hospital Hochsteiermark, Leoben, Austria; 3 Department of Psychiatry and Psychotherapeutic Medicine, Medical University of Graz, Graz, Austria; 4 Practice for General Internal Medicine, Bruck/Mur, Austria; 5 Department of Immunology and Pathophysiology, Medical University of Graz, Otto Loewi Research Center, Graz, Austria; 6 Clinical Institute of Medical and Chemical Laboratory Diagnostics, Medical University of Graz, Graz, Austria

## ⁯⁯

***Dear Editor,***

The brain-derived beta-trace protein (BTP), which is synthesized mainly in the epithelial cells of the choroid plexus, is a low molecular weight (23 - 29 kilodalton) monomeric glycoprotein of the lipocalin protein family (Blödorn et al., 1996[4]; Astor et al., 2013[2]). Two main isoforms of BTP are existing. The smaller “brain” type isoform with absent sialylation and truncated oligosaccharide chains predominates in the cerebrospinal fluid (CSF) and is rapidly cleared through the liver, whereas the larger “serum” type isoform, which predominates in serum and urine, has more fully sialylated oligosaccharide chains and is eliminated via the kidneys (Hoffmann et al., 1997[15]; White et al., 2015[31]; Enko et al., 2018[11]). The relative concentrations and functional significance of these different BTP isoforms in healthy and diseased states are not known yet (White et al., 2015[31]; Enko et al., 2018[11]). 

The messenger RNA of BTP has been detected in many human tissues, such as the central nervous system, testicular Sertoli and Leydig cells, myocardial cells, vascular endothelial cells, skin melanocytes, gastric mucosal epithelial cells, and adipocytes (White et al., 2015[31]; Orenes-Piñero et al., 2013[22]). This fact indicates that BTP may have various extracranial physiological roles in the human body (Enko et al., 2018[11]).

In recent years, BTP has been proposed as a promising novel endogenous biomarker of glomerular filtration rate (GFR) in acute and chronic kidney disease (Enko et al., 2018[11]; Leyssens et al., 2021[18]; Donadio et al., 2003[9]). Beyond its role to estimate kidney function, this brain-specific BTP with its relatively high CSF concentrations has been suggested as a sensitive and reliable marker to detect CSF leakage, which may occur in nasal secretions (rhinorrhea) or in the external auditory canal (otorrhea) (Felgenhauer et al., 1987[13]; Reiber et al., 2003[25]; McCudden et al., 2013[20]). The highly sensitive and specific BTP test for quantitative detection of CSF fistulas is also recommended in the guidelines of the European Federation of the Neurological Societies (EFNS) (Deisenhammer et al., 2009[8]).

It is well known that the CSF analysis is an essential tool for neurological diseases (Enko et al., 2020[12]). However, the physiological role of BTP in the brain and the peripheral blood has not been fully explored yet. The blood-CSF barrier regulates the permeability of molecules and agents between the bloodstream and the brain (Enko et al., 2020[12]; Engelhardt and Sorokin, 2009[10]). The CSF/serum albumin quotient (Q_ALB_) is a reliable and widely accepted surrogate marker of the blood CSF-barrier integrity (Reiber and Peter, 2001[26]). Nevertheless, the impact of blood-CSF function and acute-phase reaction on BTP concentrations in serum and CSF is not known yet.

Therefore, the present study aimed to analyze serum/CSF concentrations of BTP and albumin in patients with diagnostic lumbar punctures. Possible associations between serum and CSF BTP concentrations were assessed in individuals sub-grouped by the presence or absence of acute-phase protein and blood-CSF barrier dysfunction.

A total of 253 consecutive medical records of patients, who received diagnostic lumbar puncture with assessment for CSF and serum BTP concentrations, blood-CSF barrier function and acute-phase reaction at the Institute of Clinical Chemistry and Laboratory Medicine of the General Hospital Hochsteiermark (Leoben, Austria), were retrospectively examined. Patients < 18 years of age and individuals with acute or chronic kidney diseases were excluded. All patients provided informed consent. The ethical approval of this study was obtained from the Ethical Committee of the Medical University Graz (Graz, Austria). The study was carried out in accordance with the current version of the Declaration of Helsinki.

Venous blood and CSF samples were collected simultaneously in sterile 5 mL VACUETTE^® ^ Z Serum Clot activator tubes and 2 mL VACUETTE^® ^ Z No Additive tubes (Greiner Bio-one International GmbH, Kremsmünster, Austria). 

For quantitative determination of BTP in CSF (reference range: 8.9 - 25.9 mg/L) and serum (reference range: ≤ 0.7 mg/L), the nephelometric N Latex BTP assay (Siemens Healthineers, Erlangen, Germany) was performed on the Atellica^® ^NEPH 630 system (Siemens Healthineers). The intra- and inter-day coefficients of variation (CVs) ranged between 2.4 - 6.5 and 2.4 - 6.1 %. The serum (reference range: 35 - 52 g/L) and CSF (reference range: ≤ 350 mg/L) concentrations of albumin were also determined by nephelometric method on the Atellica^® ^NEPH 630 system (Siemens Healthineers). The intra- and inter-day CVs were between 2.7 - 3.1 and 1.7 - 3.5 %. To assess the function of the blood-CSF barrier, the Q_ALB_ was calculated (Sindic et al., 2001[28]). The upper limit of the reference range for Q_ALB _between normal and dysfunctional blood-CSF barrier was determined age-related (5.0 x 10^-3 ^for patients < 15 years, 6.5 x 10^-3 ^for patients < 60 years, and 8.0 x 10^-3 ^for patients ≥ 60 years) (Reiber et al., 2001[25]; Brettschneider et al., 2005[6]).

The C-reactive protein (CRP) and the creatinine (Jaffe) were measured on a cobas^®^ 8000 c701 analyzer (Roche Diagnostics, Rotkreuz, Switzerland). The estimated glomerular filtration rate (GFR) was calculated using the Chronic Kidney Disease Epidemiology Collaboration (CKD-EPI) equation (Levey et al., 2009[17]).

Normal distribution of the data was examined with the Kolmogorov-Smirnov test. As continuous variables were not normally distributed, they were expressed as medians with interquartile ranges (Q1 - Q3). Categorical variables were expressed as percentages. Spearman's rho (ρ) was calculated to assess possible positive or negative correlations between study parameters. Linear regression models were performed to assess the association between variables. The exact Mann-Whitney U test was calculated for subgroup comparisons of not normally distributed metric variables. A p-value < 0.05 was considered statistically significant. The statistical analyses were performed using Analyse-it® software version 4.92 (Analyse-it Software, Ltd., Leeds, United Kingdom).

Table 1(Tab. 1) shows the baseline characteristics of the study population. Of all 253 patients with diagnostic lumbar punctures, 116 (45.8 %) were male and 137 (54.2 %) were female. The average age was 49 ± 19 years. Overall, 88 (34.8 %) and 165 (65.2 %) patients were identified with and without blood-CSF barrier dysfunction. Eighty-one (32.0 %) and 172 (68.0 %) individuals were observed with (CRP > 5 mg/dL) and without (CRP ≤ 5 mg/dL) acute-phase reaction. A total of 32 (12.6 %) patients were found with combined blood-CSF barrier dysfunction and acute-phase reaction. Of all 253 patients, the median CSF and serum BTP concentrations (Q1 - Q3) were 14.7 (12.1 - 18.9) and 0.57 (0.49 -0.66) mg/L.

There was a positive correlation between serum BTP and CSF BTP (ρ = 0.265, p < 0.001), between serum BTP and age (ρ = 0.372, p < 0.001), and between serum BTP and creatinine (ρ = 0.209, p < 0.001). Serum BTP concentrations correlated also with the Q_ALB_ (ρ = 0.217, p < 0.001). There was a negative correlation between serum BTP concentrations and the CKD-EPI-GFR (ρ = -0.352, p < 0.001).

**Figure 1:**
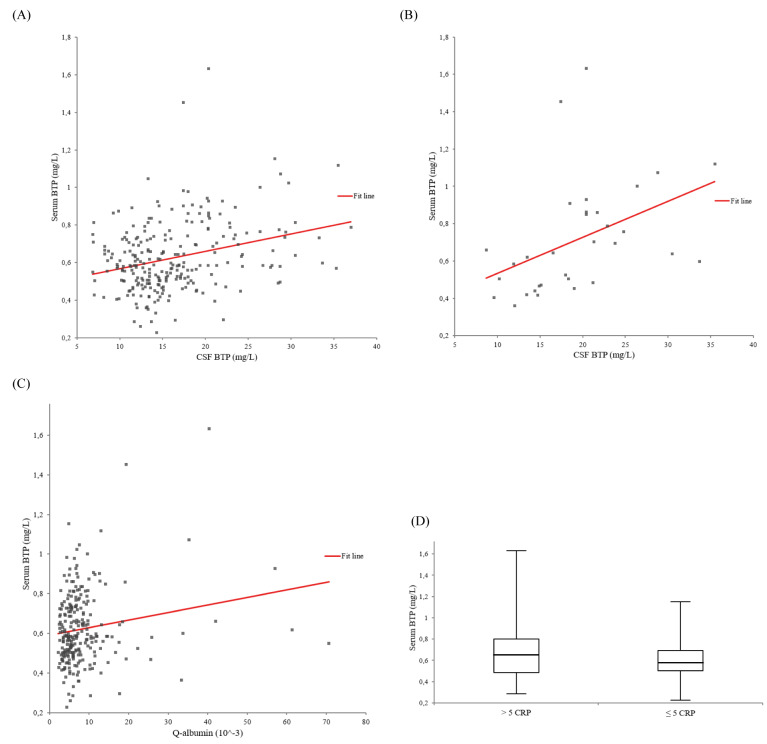
Univariate regression models between serum beta-trace protein (BTP) (adjusted for creatinine) and cerebrospinal fluid (CSF) BTP. A. In all 253 patients: ß-coefficient = 0.294, p < 0.001. B. In 32 individuals with blood-CSF barrier dysfunction and acute-phase reaction: ß-coefficient = 0.428, p = 0.015. C. Univariate regression model between serum beta-trace protein (BTP) (adjusted for creatinine) and the cerebrospinal fluid (CSF)/serum albumin quotient (Q_ALB_) in all 253 patients. ß-coefficient = 0.170, p < 0.007. D. Serum beta-trace protein (BTP) and acute-phase reaction. Box-and-whisker plots of serum BTP concentrations between 81 and 172 individuals with (CRP > 5 mg/L) and without (CRP ≤ 5 mg/L) acute-phase reaction (p = 0.046). The central boxes represent the 25^th^ to 75^th^ percentile range. The lines inside the boxes show the median value for each group. Minimum and maximum are indicated as whiskers with end caps.

The univariate regression models between serum and CSF BTP are presented in Figure 1(Fig. 1). In all 253 patients, liquor BTP showed a statistically relevant influence on serum BTP (ß-coefficient = 0.294, p < 0.001) (Figure 1A(Fig. 1)). In 32 individuals with blood-CSF barrier dysfunction and acute-phase reaction, the association between serum and CSF BTP was much stronger with a markedly higher ß-value (ß-coefficient = 0.428, p = 0.015) (Figure 1B(Fig. 1)) compared to 116 individuals without blood-CSF barrier dysfunction and acute-phase reaction (ß-coefficient = 0.221, p = 0.017). As shown in Figure 1C(Fig. 1), the Q_ALB _had a statistically relevant influence on the serum BTP concentration (ß-coefficient = 0.170, p < 0.007).

As demonstrated in Figure 1D(Fig. 1), 81 patients with an acute-phase reaction (CRP > 5 mg/L) had significantly higher median (Q1 - Q3) serum BTP concentrations (0.65 (0.49 - 0.80) vs. 0.58 (0.50 - 0.69) mg/L, p = 0.046) compared to 172 individuals without acute-phase reaction (CRP ≤ 5 mg/L). Individuals with acute-phase reaction showed also higher CSF BTP levels (16.5 (12.0 - 21.2) vs. 14.5 (12.1 - 17.8) mg/L, p = 0.085).

In the present study, serum and CSF BTP concentrations were assessed at the blood-CSF barrier in 253 CSF/serum pairs obtained from clinical routine. The serum BTP levels were positively correlated with the CSF BTP levels and in the linear regression model CSF BTP showed a significant influence on serum BTP. Patients with acute-phase reaction and blood-CSF barrier were observed with a much stronger association between serum and CSF BTP compared to individuals without acute-phase reaction and normal blood-CSF barrier function. 

Herein, the median CSF and serum BTP concentrations of 253 patients were 14.7 (12.1 - 18.9) and 0.57 (0.49 - 0.66) mg/L. These results are in line with a previous study by Tumani et al., who measured mean CSF and serum BTP concentrations of 14.6 ± 4.6 and 0.46 ± 0.13 mg/L in 40 adult control patients (Tumani et al., 1998[30]). In the present study, serum BTP concentrations positively correlated with the CSF/serum albumin ratio (Q_ALB_) and age. Albumin- and age-related increase of CSF BTP concentrations were also reported by Tumani et al. (Tumani et al., 1998[30]). A positive correlation between serum BTP and Q_ALB _was also confirmed by Nilsson et al., who investigated CSF and serum BTP samples of 21 patients scheduled for knee arthroplastic surgery (Nilsson et al., 2010[21]).

Here, we observed higher serum BTP and CSF BTP levels in individuals with an acute-phase reaction compared to subjects without acute-phase reaction. These results indicate that inflammation may influence the BTP status in the brain and in the blood circulation. A previously published study reported that the choroid plexus, which synthesizes the BTP, is a relevant mediator of immune signals between the peripheral blood and the brain (Marques et al., 2007[19]). An acute-phase reaction may influence the expression of choroid plexus proteins, which leads to increased CSF BTP levels (Marques et al., 2007[19]). Another previous study showed that in 18 patients with an acute inflammatory demyelinating polyneuropathy the BTP concentrations were significantly increased in the CSF compared to 18 controls (Huang et al., 2009[16]).

An acute-phase reaction may not only influence the BTP production in the choroid plexus but also the CSF flow rate (Huang et al., 2009[16]). CSF BTP concentrations are strongly influenced by the topographical localization in the central nervous system (Tumani and Brettschneider, 2005[29]). A previous report about neuroprotein dynamics in the CSF showed that lumbar BTP concentrations were higher compared to the ventricular BTP (Brandner et al., 2013[5]). The normal mean BTP concentration increases from 1.5 mg/L in the ventricular CSF to 16.6 mg/L in the lumbar CSF (Reiber, 2003[24]). The increasing BTP concentration between ventricular and lumbar CSF and the CSF flow rate are the most relevant factors influencing BTP concentrations in CSF (Reiber, 2001[23]). Changes in the production and dynamics of BTP in the CSF could be one explanation for higher CSF BTP levels in individuals with acute-phase reaction found in this study.

A previous study, which investigated CSF and serum BTP concentrations in 35 patients before and after elective knee replacements, could not found any significant BTP changes during and after the surgery (0.66 ± 0.23 vs. 0.63 ± 0.23 mg/L, p = 0.189) (Anckarsäter et al., 2007[1]). A recent study, comprising 99 patients with liver cirrhosis and 99 matched controls, showed that serum BTP concentrations are not affected by hepatic dysfunction (Chakraborty et al., 2018[7]). However, present results demonstrate that acute-phase reaction and blood-CSF barrier dysfunction affects the BTP status. The highly specific expression of BTP at the blood-CSF barrier indicates a potential role of this biomarker in transport, maturation and maintenance of this physiological barrier (Hoffmann et al., 1996[14]). Recently, Baranyi et al. reported that BTP might have the potential to serve as a new non-invasive biomarker to indicate impairment of the blood-brain barrier integrity (Baranyi et al., 2017[3]).

The strength of this study is the high number of consecutive serum/CSF BTP pairs obtained from neurological patients out of daily clinical routine. Nevertheless, some limitations of this work should be addressed: the data do not allow to draw conclusions about possible links between CSF or serum BTP concentrations and neurological conditions. Because of CSF dynamics of brain-derived proteins, the exact relationship of the lumbar BTP, where CSF samples are collected, and the site of BTP synthesis in the brain is not fully possible to evaluate (Reiber, 2001[23]; Nilsson et al., 2010[21]).

Furthermore, Serum/CSF BTP concentrations were measured at one single time point. Therefore, the intra-patient variability of BTP levels was not available.

Significant association of serum and CSF BTP concentrations was observed in 253 diagnostic serum/CSF pairs of clinical routine. Patients with acute-phase reaction and blood-CSF barrier dysfunction had a much stronger association with higher serum and CSF BTP levels compared to individuals without acute-phase reaction and blood-CSF barrier dysfunction. Acute-phase reaction has a substantial impact on the BTP status.

## Conflict of interest

The authors declare that there is no conflict of interest.

## Acknowledgements

Cost for BTP reagents were reimbursed by Siemens Healthineers (Vienna, Austria).

## Figures and Tables

**Table 1:**
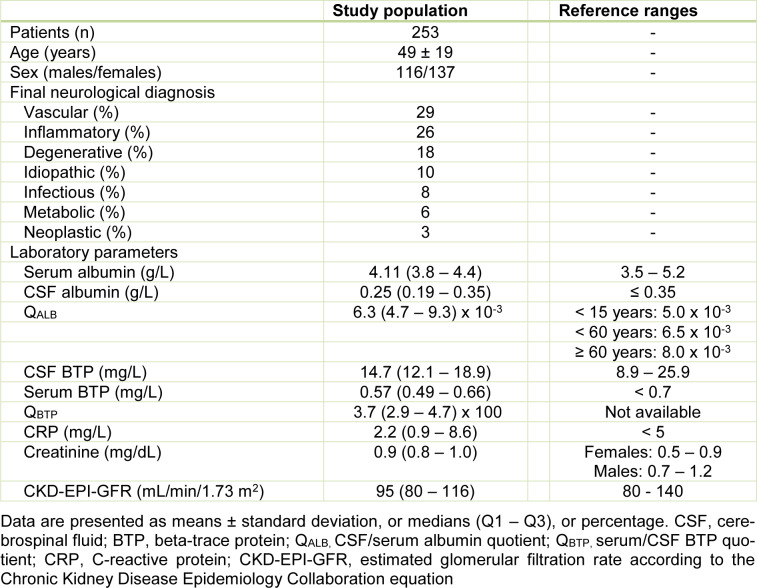
Baseline characteristics of the study population
